# Supplementary material for: The Multilateral Efficacy of Chitosan and Trichoderma on Sugar Beet
Source: J Fungi (Basel). 2022 Jan 29;8(2):137. doi: 10.3390/jof8020137 (PMC8879458; doi:10.3390/jof8020137)
Supplement: Supplementary file 1 [file jof-08-00137-s001.zip › jof-1532344-supplementary.pdf]

Journal of *Fungi*

Supplementary Information

The Multilateral Efficacy of Chitosan and *Trichoderma* on Sugar Beet

Lisa Kappel [1, 2], Nicole Kosa [2] and Sabine Gruber [1, 2,*]

[1] Department of Microbiology, University of Innsbruck, 6020 Innsbruck, Austria; lisa.kappel@uibk.ac.at
[2] Department of Bioengineering, FH Campus Wien, University of Applied Sciences, 1190 Vienna, Austria; nicole.kosa@stud.fh-campuswien.ac.at
* Correspondence: sabine.gruber@fh-campuswien.ac.at

Appendix A: Supplementary tables

Supplementary Table S1: RT-PCR primers for *T. atroviride* used in this study

pID (JGI)[1]	gene	primer name	Sequence (5′- 3′)	number of cycles	source
28913	*cda1*	Ta_cda1_RTs	TCCTCCATGCAGGACTCTGA	30	this study
		Ta_cda1_RTa	GCTTGGAGCAGCAGTTTTTGT		this study
147996	*cda2*	Ta_cda2_RTs	CAAAGTCATTGCCCTGTCGC	30	this study
		Ta_cda2_RTa	AGACGTCACTAGGTCCGTGA		this study
78914	*cda3*	Ta_cda3_RTs	CACTCGTCCGTCATAGAGGC	30	this study
		Ta_cda3_RTa	CCACAGCGTCTTCCAATTGC		this study
291124	*cda4*	Ta_cda4_RTs	ACCACCAAAAGGGAGTGTCG	30	this study
		Ta_cda4_RTa	ACCGGCCATTGGAGGATTTC		this study
292288	*cda5*	Ta_cda5_RTs	CAACTCCTCGACGTGTTGGA	30	this study
		Ta_cda5_RTa	GTGCAAGTAGCTGTCGGTCT		this study
142446	*cda6*	Ta_cda6_RTs	CTGCCAGGCCTACTATCTGC	30	this study
		Ta_cda6_RTa	TCATGCGTCCTTTCGTAGCA		this study
54365	*cho1*	Ta_cho1_RTs	GCTCCAACAAACTCTCGGGA	30	this study
		Ta_cho1_RTa	TTATCGCCGCTCAGACCATC		this study
80199	*cho2*	Ta_cho2_RTs	GCGACATTCCCAATGCCATC	30	this study
		Ta_cho2_RTa	GCTGTTCTCAAAATCCGCCG		this study
145108	*cho3*	Ta_cho3_RTs	TATGGCGAGACAGCGTTCAA	30	this study
		Ta_cho3_RTa	CCGCTTGAGGTTCCAAGACT		this study
197013	*cho4*	Ta_cho4_RTs	GCGGTCTTCCAAATGCCATT	30	this study
		Ta_cho4_RTa	CAGGCTGGTAACGGGTTGAT		this study
216890	*cho5*	Ta_cho5_RTs	ACCATCACCAGCTACAAGGC	30	this study
		Ta_cho5_RTa	ATGCAGGAGGAGGAGGAGAG		this study
16857	*cho6*	Ta_cho6_RTs	GTAGCAGACCTGGCTGGAAG	30	this study
		Ta_cho6_RTa	GAAGCACCAGCACAATGTCC		this study
131598	*chi18-5*	Ta_chi18-5_RTs	GTCTCTGGAGATGCCTACGC	30	this study
		Ta_chi18-5_RTa	AGTCGAGAACTTGGCCAAGG		this study
136120	*nag1*	Ta_nag1_RTs	GAGCGATGTCCTACAGCCTC	30	this study
		Ta_nag1_RTa	GGGCCAGATGATGTTGTCCA		this study
299956	*gfa1*	Ta_gfa1_RTs	TCATCGAGGGTCTTGGCAAC	30	this study
		Ta_gfa1_RTa	TCGACAGTGACAGACTTGGC		this study

302952	*epl1*	Ta_epl1_RTa	CTTGCTCTCTTCACCGCCGC	27	[1]
		Ta_epl1_RTa	CCGTTGGTCAGGGCATTCAT		[1]
300828	*tef1*	Ta_tef1_RT2s	CGACATTGCCCTCTGGAAGT	20	this study
		Ta_tef1_RT2a	ATCCTGAAGGGGAAGACGGA		this study

cda…predicted chitin deacetylase, *cho*…predicted chitosanase, *chi18-5*… endochitinase, *nag1*… N-acetylglucosaminidase, *gfa1*… predicted glucosamine-fructose-6-phosphate aminotransferase, *epl1*…ceratoplatanin, tef1… translation elongation factor as housekeeping gene [1]https://mycocosm.jgi.doe.gov/Triat2/Triat2.home.html .

Supplementary Table S2: RT-qPCR primers for *B. vulgaris* used in this study

**GenBank identifier/ reference sequence**[1]	**gene**	**primer name**	**Sequence (5´- 3´)**	**source**
GQ375163.1	DMRL	qRT_Bvulg_DMRLf	TCCGCCTCCTCTGGAATACT	this study
		qRT_Bvulg_DMRLr	CGCTGCCTCAGAACCCTTAT	this study
X75946.1	GLU	qRT_Bvulg_GLUf	CAACTTACCTTCCGAGGAAG	[2]
		qRT_Bvulg_GLUr	CTCTAACCGCTTGGAGGGTC	this study
S66038.1	SE2	qRT_Bvulg_SE2f	ATTGTCATATACTGGGGCC	[2]
		qRT_Bvulg_SE2r	TTGTAGCAGGGTCACAGTGC	this study
BI073261.1	GST	qRT_Bvulg_GSTf	GCTTTGGATTTGTGGACATTGC	this study
		qRT_Bvulg_GSTr	TCGGACACTCTGCCTCTACA	this study
BQ585675.1	PAL	qRT_Bvulg_PALf	GAAGATCGGAGCCTTCGAGG	this study
		qRT_Bvulg_PALr	TTACCACTCTCGTAGGCAGC	this study
BQ593253.1	AAT	qRT_Bvulg_AATf	GCAAGACACCCACGACTTCT	this study
		qRT_Bvulg_AATr	AGCAAACGCCTTGGAAAAGC	this study
XM_010676890.2	PR-3	qRT_Bvulg_PR-3f	CACTGGAGGATGGGCTACTG	this study
		qRT_Bvulg_PR-3r	CCACCCTCCAACGATGACAT	this study
XM_010679634.2	GAPDH	qRT_Bvulg_GAPDHf	CATCAAGGCGGAATCAGAAGG	[3]
		qRT_Bvulg_GAPDHr	ACGAGCTTTGCGAAGTGGTC	[3]
XM_010671243.2	TUB1	qRT_Bvulg_TUBf	CTATGCATCTTCACTTTGAAACAGTTTT	[4]
		qRT_Bvulg_TUBr	CTAGAAGAGGCTGACGAGAAAGAAG	[4]
XM_010673056.2	ACT7	qRT_Bvulg_ACT7f	GGCAAACAGGGAAAAGATGA	this study
		qRT_Bvulg_ACT7r	GGCTCACACCATCACCAGAA	this study

GLU… predicted glucanase, SE2/PR-3… predicted chitinases, AAT… predicted aspartate aminotransferase, PAL… predicted phenylalanine ammonia lyase, GST… predicted glutathione-S-transferase, DMRL… predicted 6,7-dimethyl-8-ribityl lumazine synthase; housekeeping genes: GAPDH… predicted glyceraldehyde-3-phosphate dehydrogenase, TUB1… predicted tubulin, ACT7… predicted actin.

[1]https://www.ncbi.nlm.nih.gov/nuccore/

Supplementary Table S3: Expression analysis of indicator genes in *B. vulgaris* corresponding to Figure 4D.

	**0.1% HMW CHSN**		***T. atroviride* spores**	
**gene #**	**t1**	**t2**	**t1**	**t2**
GLU	0.387[a]	0.553	0.206[b]	0.577
SE2	0.922	0.926	0.411[b]	0.873
PR-3	1.742[a]	1.328	1.985[b]	1.785[b]
AAT	1.422[a]	0.991	1.081	1.261
PAL	1.385[b]	1.574[b]	1.093	1.157
GST	1.209[b]	1.966[c]	0.964	0.992
DMRL	1.264[b]	0.861	1.054	0.823

Statistical significance indicated with: a, $p < 0.001$; b, $p < 0.01$; c, $p < 0.05$

Supplementary Table S4: Expression analysis of indicator genes in *B. vulgaris* seedlings corresponding to Figure 5.

	**seeds coated 1% HMW CHSN**		**seeds coated *T. atroviride* spores**		**uncoated phytagel with 0.1% HMW CHSN**	
**gene #**	**SHOOT**	**ROOT**	**SHOOT**	**ROOT**	**SHOOT**	**ROOT**
PR-3	0.732	1.030	2.471[c]	2.614	3.882[a]	5.988[c]
GLU	1.117	0.889	2.953	5.430	1.504	7.753[b]
SE2	0.854	1.695	3.368[b]	7.211[b]	0.340[c]	4.215[b]
PAL	0.891	0.963	1.022	1.266	0.544[c]	1.415
AAT	1.192	1.356	2.420[a]	2.945[a]	1.051	2.409[b]
DMRL	1.317[a]	1.061	1.931[a]	1.693[a]	1.427[a]	1.390[b]
GST	1.048	1.349	3.123[b]	1.998[b]	0.533[c]	3.122[b]

Statistical significance indicated with: a, $p < 0.001$; b, $p < 0.01$; c, $p < 0.05$

Appendix B: Supplementary figures

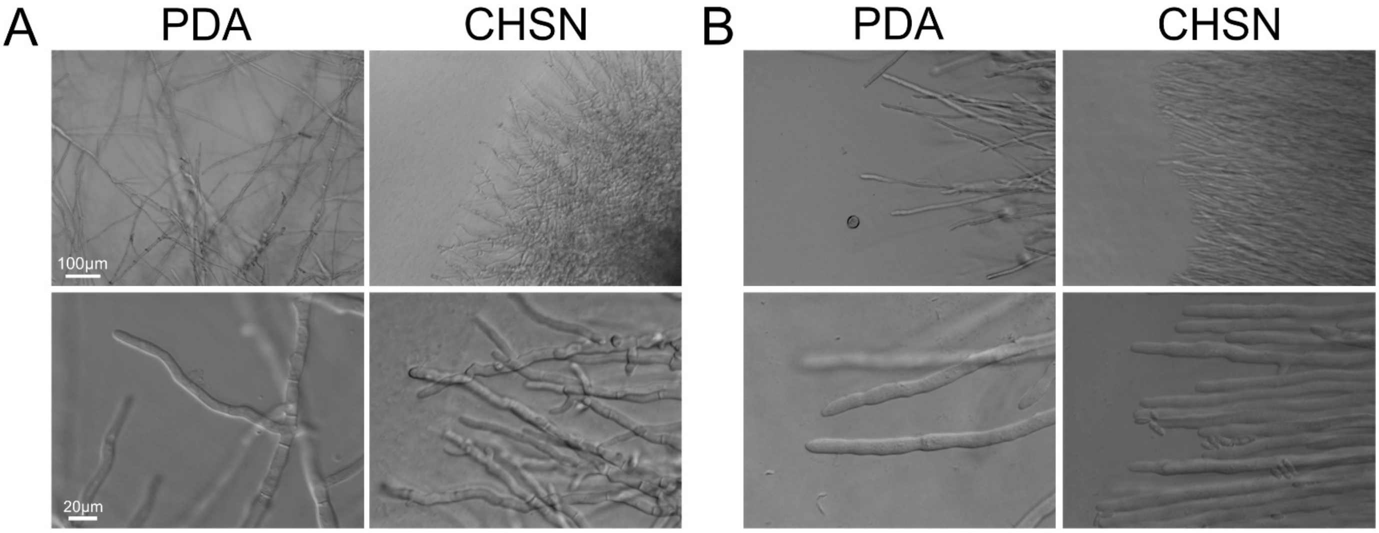

Supplementary Figure S1: Hyphal agglomeration of *C. beticola* and *F. oxysporum* on chitosan. Microscopic analysis of *C. beticola* (A) and *F. oxysporum* (B) on PDA or PDA supplemented with 0.6 mg ml^{-1} LMW chitosan after growth for 120 h and 48 h, respectively. Scale bars are indicated.

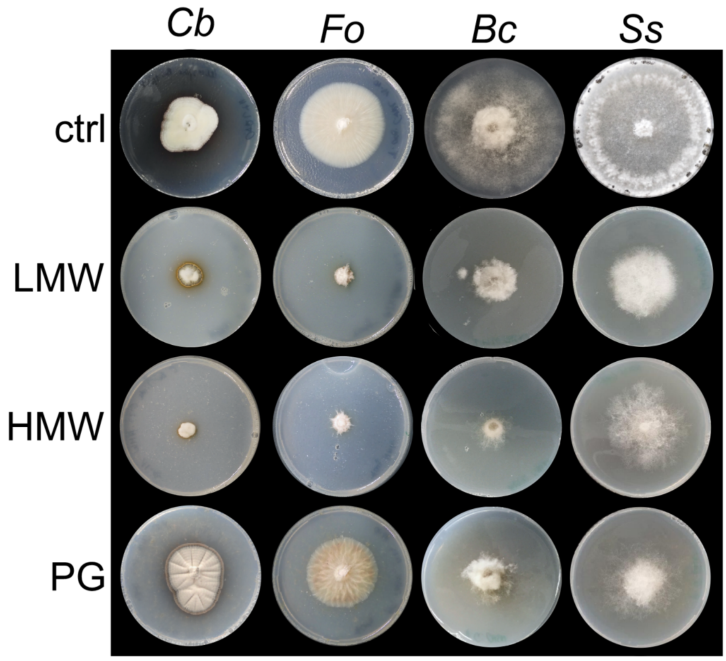

Supplementary Figure S2: Growth of pathogens on chitosans. Figure corresponds to Figure 2 in the manuscript, showing growth of the pathogens in absence of confrontation with *T. atroviride*. Strains were cultured on (PDA, ctrl) or supplemented with 2 mg ml^{-1} LMW, low molecular weight; HMW, high molecular weight; PG, practical grade chitosan. *Cb, C. beticola; Fo, F. oxysporum; Bc, B. cinerea; Ss, S. sclerotiorum*

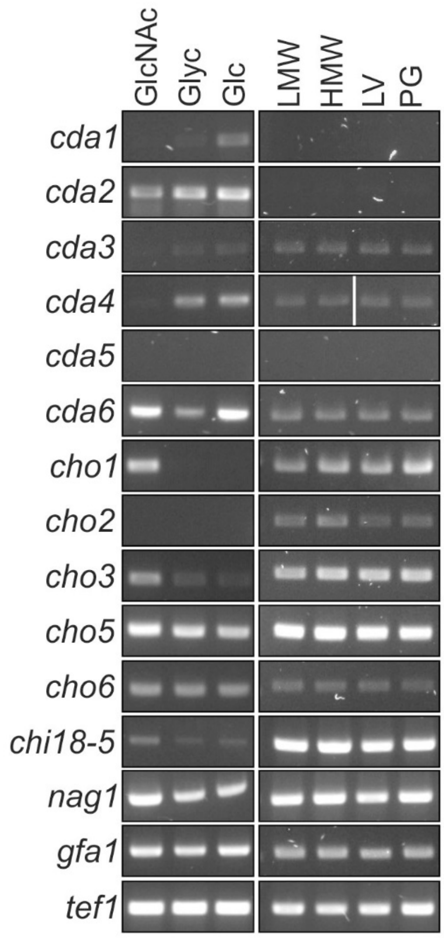

Supplementary Figure S3: Differential expression of mycoparasitism related genes in *T. atroviride* in response to growth on chitosan. RT-PCR was performed from 24 h cultures of *T. atroviride* grown in liquid minimal medium supplemented with 10 mg ml^{-1} of the respective carbon source: *N*-acetylglucosamine (GlcNAc), glycerol (Glyc), glucose (Glc), low molecular weigh (LMW), high molecular weight (HMW), low viscous (LV), practical grade (PG) chitosan. The expression of the indicated genes was analysed by use of the RT-PCR primer pairs listed in the Supplementary Table S1.

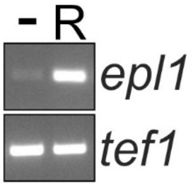

Supplementary Figure S4: *T. atroviride* communicates with *Beta vulgaris* when colonizing its roots. RT-PCR was performed from RNA extracted from *T. atroviride* colonizing 10 day old seedlings from *in vitro* culture and compared to *T. atroviride* cultured on cellophane as surface. The expression of the indicated genes was analysed by use of the RT-PCR primer pairs listed in the Supplementary Table S1.

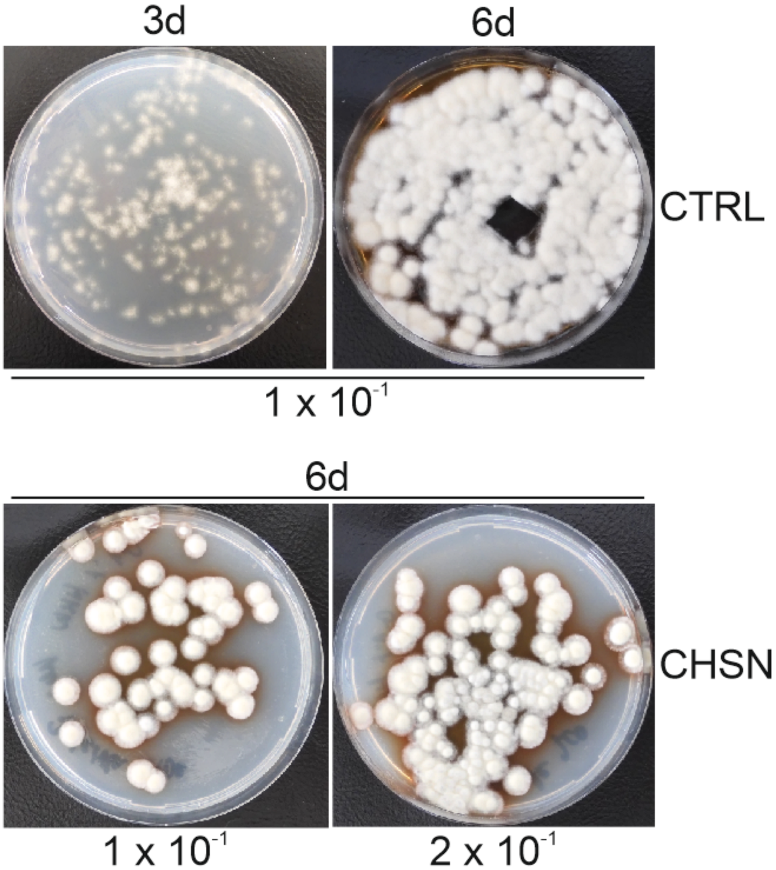

Supplementary Figure S5: *C. beticola* colony formation from single spores. Single spores of *C. beticola* were spread on PDA plates containing 1mg ml^{-1} HMW chitosan (CHSN) and incubated for 6 days (6d) at 25°C in the dark. PDA plates served as control (CTRL) and were incubated for 3 (3d) and 6 (6d) days. A 1 x 10^{-1} dilution of the spore solution is shown for the CTRL condition and a 1 x 10^{-1} and 2 x 10^{-1} dilution are shown for CHSN.

References

1. Gaderer, R. Functional characterization of a fungal gene family encoding plant defense response-eliciting proteins in *Trichoderma* biocontrol species. Master thesis, TU-Wien, Vienna, **2012**.
2. Takenaka, S.; Tamagake, H. Foliar spray of a cell wall protein fraction from the biocontrol agent *Pythium oligandrum* induces defence-related genes and increases resistance against Cercospora leaf spot in sugar beet. *J. Gen. Plant Pathol.* **2009**, *75*, 340-348, doi:10.1007/s10327-009-0186-9.
3. Schmidt, J.; Dotson, B.R.; Schmiderer, L.; van Tour, A.; Kumar, B.; Marttila, S.; Fredlund, K.M.; Widell, S.; Rasmusson, A.G. Substrate and plant genotype strongly influence the growth and gene expression response to *Trichoderma afroharzianum* T22 in sugar beet. *Plants (Basel)* **2020**, *9*, 1-14, doi:10.3390/plants9081005.
4. Stevanato, P.; Broccanello, C.; Moliterni, V.; Mandolino, G.; Barone, V.; Lucini, L.; Bertoldo, G.; Bertaggia, M.; Cagnin, M.; Pizzeghello, D. Innovative approaches to evaluate sugar beet responses to changes in sulfate availability. *Front. Plant Sci.* **2018**, *9*, 1-9, doi:10.3389/fpls.2018.00014.
